# Transcriptomic Analysis of Flower Bud Differentiation in *Magnolia sinostellata*

**DOI:** 10.3390/genes9040212

**Published:** 2018-04-16

**Authors:** Lijie Fan, Mengqian Chen, Bin Dong, Ninghang Wang, Qin Yu, Xingli Wang, Lingjuan Xuan, Yaling Wang, Shouzhou Zhang, Yamei Shen

**Affiliations:** 1 College of Landscape and Architecture, Zhejiang Agriculture and Forestry University, Hangzhou 311300, China; ffflj0311@163.com (L.F.); mengqian818@163.com (M.C.); maddonbin@gmail.com (B.D.); wangninghang789@163.com (N.W.); Celery221@126.com (Q.Y.); wxlmayday1992@163.com (X.W.); 18296157009@163.com (L.X.); 2 Xian Botanical Garden of Shaanxi Academy of Science, Xian 710061, China; wangyl100@aliyun.com; 3 Shenzhen Fairy Lake Botanical Garden, Shenzhen 518004, China; shouzhouz@126.com

**Keywords:** flower bud differentiation, flowering gene, RNA-seq, qRT-PCR, *Magnolia* sp.

## Abstract

Magnolias are widely cultivated for their beautiful flowers, but despite their popularity, the molecular mechanisms regulating flower bud differentiation have not been elucidated. Here, we used paraffin sections and RNA-seq to study the process of flower bud differentiation in *Magnolia sinostellata*. Flower bud development occurred between 28 April and 30 May 2017 and was divided into five stages: undifferentiated, early flower bud differentiation, petal primordium differentiation, stamen primordium differentiation, and pistil primordium differentiation. A total of 52,441 expressed genes were identified, of which 11,592 were significantly differentially expressed in the five bud development stages. Of these, 82 genes were involved in the flowering. In addition, MADS-box and *AP2* family genes play critical roles in the formation of flower organs and 20 differentially expressed genes associated with flower bud differentiation were identified in *M. sinostellata*. A qRT-PCR analysis verified that the MADS-box and *AP2* family genes were expressed at high levels during flower bud differentiation. Consequently, this study provides a theoretical basis for the genetic regulation of flowering in *M. sinostellata*, which lays a foundation for further research into flowering genes and may facilitate the development of new cultivars.

## 1. Introduction

Flower bud differentiation is an important stage in angiosperm development. The vegetative meristem is transformed into the floral meristem, which forms the base of the floral organ before developing into the floral tissues. The complex process of floral development arises in response to the integration of signals from the external environment and internal factors [1]. In *Arabidopsis thaliana*, flower development was found to involve six pathways; the photoperiod, gibberellin, vernalization, autonomous, senescence, and ambient-temperature pathways [2]. These pathways co-regulate the flower-specific genes, causing the vegetative meristem to physiologically transition into the floral meristem. Many genes have been found to integrate the signals received via these pathways, such as *FT* (*FLOWER LOCUS T*) and *SOC1* (*SUPPRESSOR OF OVEREXPRESSION OF CO 1*), both of which promote floral development and lead to early flowering in plants [3,4]. *LFY* (*LEAFY*) integrates the signals of multiple floral induction pathways and activates floral organ development [5,6]. These integrative genes are highly conserved in plants. The *FT* genes of apple (*Malus × domestica*) and longan (*Dimocarpus longan*) influenced flowering when heterologously expressed in *Arabidopsis* [7,8], while the *Arabidopsis FT* and *LFY* genes regulated flowering when heterologously expressed in the satsuma mandarin (*Citrus unshiu*) [9] and poplar (*Populus* sp.) [10].

Flower development is controlled by floral organ trait genes. The classic ABC model of floral organ development, developed by Meyerowitz and Coen [11,12], proposes that class A genes control the formation of the sepals and petals, class B genes control the formation of the petals and stamens, and class C genes control the formation of the stamens and carpels. Members of these gene categories have been cloned from a variety of plants [13]. Later studies found that *FBP11* (*FLORAL BINDING PROTEIN11*) controls ovule development in *Petunia* sp. [14], while other positive regulators of many aspects of floral development, such as *SEP* (*SEPALLATA*), were identified in *Arabidopsis* sp. and other model plants [15]. Thus, the ABC model was replaced by the ABCDE model, which states that A- and E-class genes determine the first whorl, the calyx; A-, B- and E-class genes interact to form the petals in the second whorl; B-, C-, and E-class genes interact to regulate the third whorl, the stamens; C- and E-class genes control carpel development in the fourth whorl; and D- and E-class genes are involved in the formation of the ovary [16,17]. The floral development mode is composed of the activities of MADS-box and *AP2* family transcription factors [13,18]. *AP2* gene plays a role as A-class genes in a flower development model and MADS-box transcription genes participate in various types [13].

Magnolias (*Magnolia* sp.) are widely distributed shrubs and trees that produce beautiful flowers in the spring. The Magnoliaceae have long been considered an ancient angiosperm family and much of the current research on members of this family focuses on their systematic evolution [19,20]. The studies of Magnoliaceae flower bud differentiation mostly involve morphological observation [21], leaving the molecular mechanisms underpinning their flower development largely unclear. Homologs of *AP3* (*APETALA3*) and *AG* (*AGAMOUS*) have been cloned from the flower buds of *Magnolia wufengensis* and their expression patterns have been reported [22,23], while other studies investigated the transcriptomic regulation of petal color in *Magnolia sprengeri* [24] and the biosynthetic pathways regulating floral volatile organic compounds in *Magnolia champaca* [25].

*Magnolia sinostellata* is an endangered magnolia shrub species with high ornamental value [26]. Its morphological features are similar to *Magnolia stellata* [27]; however, the two species can be differentiated by their twig color, by staining during meiosis [26], and because *M. sinostellata* blooms earlier than *M. stellata*. The flowers of *M. sinostellata* wither in late March and the leaves begin to grow nearly a month later. During the process of flower bud differentiation, the outer bud sequentially forms two to three layers of shallow, brown, spathe-like bracts. Flowers develop once a year, beginning at the end of April and ending around the beginning of June, for a total differentiation period of almost 40 days.

We have previously investigated flower bud differentiation in *Magnolia biondii*, *Magnolia denudata* and *Magnolia × soulangeana* ‘Red lucky’ [28]; however, the molecular regulation of flower bud differentiation has not been previously reported for *Magnolia* sp. Here, we explored the morphological characteristics of flower bud differentiation in *M. sinostellata* and performed a transcriptomic analysis to identify genes that were differentially transcribed at the five floral bud stages. The results of this study provide a foundation for future studies and breeding efforts focusing on flowering in *Magnolia* sp.

## 2. Materials and Methods

### 2.1. Plant Materials

The *M. sinostellata* individuals (approximately 10 years old) used in this study are maintained outdoor of the nursery in Zhejiang Agriculture and Forestry University (30°15′14″ N and 119°43′39″ E). The subtropical monsoon climate is warm and humid, with sufficient light and an annual average precipitation of approximately 1600 mm. All mature *M. sinostellata* trees were managed according to ordinary culture practices.

### 2.2. Morphological Observation

A total of 15 flower buds were collected from the middle and upper regions of the tree crown every month from June 2016 to June 2017 and were stored in FAA fixative (8:1:1 ratio of 50% ethanol: formaldehyde: glacial acetic acid). The initiation of a new leaf, and the flower buds were collected every seven days and were observed after the flower bud began to differentiate. Additionally, the sampling time was shortened to every three days until the end of differentiation. The experiment had three biological replications, and each replication had 10 buds. The development process was observed using an anatomical microscope [29]. Samples were sliced to a 10-μm thickness and subjected to safranin fast green staining for 30 s. The samples were sealed with neutral balata and then observed and photographed using an Axio Imager A2 positive fluorescence microscope (Carl Zeiss, Oberkochen, Germany).

### 2.3. RNA Extraction and cDNA Library Construction

Total RNA was extracted from flower buds at the same stages as those observed above using an RNAprep Pure Plant Kit (TaKaRa, Dalian, China), with three biological replicates at each stage. The quality and purity of the RNA samples were assessed using an RNA 6000 Nano LabChip Kit and a Bioanalyzer 2100 (Agilent Technologies, Santa Clara, CA, USA), using an RNA integrity number (RIN) of >7.0. Poly-(A)-containing mRNA was purified using oligo (dT) magnetic beads and an Oligotex mRNA Kit (Qiagen, Hilden, Germany). Fragmentation buffer was added to disrupt the mRNA strands into short fragments, which were used as templates to synthesize the first-strand cDNA using reverse transcriptase and random hexamer primers. The second-strand cDNA was synthesized using buffer, dNTPs, RNase H, and DNA polymerase I. The double-stranded cDNA fragments were subjected to end repair and adapter ligation. Adapter-modified fragments were selected using gel purification and PCR amplified to create the final cDNA library.

### 2.4. Illumina Sequencing, Assembly and Annotation

The cDNA library was sequenced on an Illumina HiSeq 4000 sequencing platform (Illumina, San Diego, CA, USA) by LC Sciences (Hanhzhou, China) to yield 2 × 150-bp paired-end raw reads. The sequenced raw reads were subjected to a quality check using FastQC [30]. The adapter sequences were removed from the raw reads. Reads with a ratio of ambiguous *N* nucleotides greater than 5% and those with low-quality sequences (quality score of less than 20) were removed. Sequencing reads were de novo assembled using Trinity software under default parameters and with a *k*-mer size of 25 [31]. The transcriptomes were assembled using pooled reads from all replications and stages. Assembly quality was critically assessed by LC Sciences Company (http://www.lc-bio.com/) before subsequent analyses. The assembled transcriptome sequences were named ‘unigenes’.

All unigenes were queried against six commonly used databases using BLASTx search to identify homologs (*E*-value < 10^–10^). The databases used were Swiss-prot [32], Nr [33], KEGG [34], KOG [35], Pfam [36], and GO [37].).

### 2.5. Differential Gene Expression Analysis

To analyze the differentially expressed genes (DEGs) during flower bud differentiation, the number of reads of each of the contigs was converted to reads per kilobase per million (RPKM) using RSEM 1.2.31 [38]. DESeq was used to determine the FDR (false discovery rate) threshold. If the FDR was less than 0.05 in the multi-group comparison, it was considered to be a significantly different expression level. The DEGs were further annotated with GO functional and KEGG pathway analyses using the software TBtools [39]. *p*-values generated from the enrichment analyses were subjected to multiple hypotheses testing by PerlScript [40], and a *p*-value of <0.05 considering statistically significant. Furthermore, the DEGs were annotated by a BLAST search against known flowering genes in *Arabidopsis* sp. [16,41] and then screened for functions known to be involved in floral development.

### 2.6. qRT-PCR Analysis

Total RNA was extracted from flower buds at the five developmental stages. After treatment with DNase, the total RNA was reverse transcribed to produce cDNA using a reverse transcription system with PrimeScript™ RT Master Mix (Perfect Real Time, TaKaRa, Dalian, China). qRT-PCR was conducted using a Light Cycler 480II (Roche Applied Science, Penzberg, Germany). Each 20-µL qRT-PCR reaction contained 10 µL SYBR Premix Ex Taq^TM^II (TaKaRa, Dalian, China), 2 µL cDNA (80 ng/µL), 0.8 µL forward primer (10 μM), 0.8 µL reverse primer (10 μM) and 6.4 µL H_2_O. The primers were designed using Primer 5.0 (Table S1). The amplification program was as follows: 30 s at 95 °C, followed by 40 cycles of 5 s at 95 °C and 30 s at 60 °C. All qRT-PCR experiments were conducted in triplicate with three biological replicates. *EF-1α* was used as the reference gene (Table S2, Figure S1) and the relative transcript abundances were calculated using the 2^−∆∆*C*t^ method [42].

## 3. Results

### 3.1. Morphological Changes during the Different Stages of Flower Bud Differentiation

**Figure 1:**
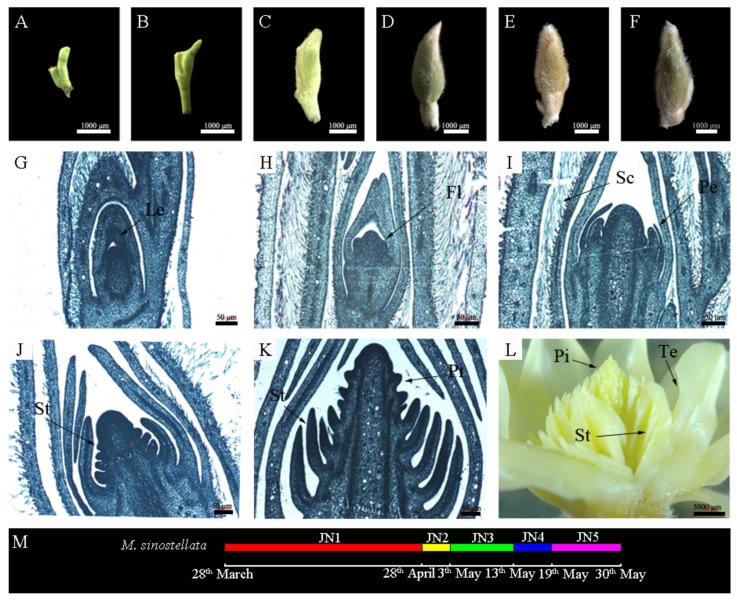
Morphological characteristics of the flower bud differentiation process in *Magnolia sinostellata.* (**A**,**G**) Undifferentiated stage (JN1); (**B**,**H**) Early flower bud differentiation stage (JN2); (**C**,**I**) Petal primordium differentiation stage (JN3); (**D**,**J**) Stamen primordium differentiation stage (JN4); (**E**,**K**) Pistil primordium differentiation stage (JN5); (**F**) The fully developed flower bud; (**L**) A dissected flower bud; (**M**) A timeline of flower bud differentiation. Le: leaf; Fl: Flower primordium; Sc: Scales; Pe: Petal primordium; St: Stamen primordium; Pi: Pistil base; Te: Tepal.

Based on the morphological features of floral bud differentiation in *M. sinostellata,* we divided the process into the following five stages (Figure 1): undifferentiated (JN1), early flower bud differentiation (JN2), petal primordium differentiation (JN3), stamen primordium differentiation (JN4), and pistil primordium differentiation (JN5).

In the undifferentiated stage, the buds were yellowish green, smooth on the outside, and lacked scale hairs. The tip of the bud was pointed and clung to the petiole of the neighboring leaf (Figure 1A). The growth point of the flower bud was small and the differentiating primordium cells were small and closely arranged (Figure 1G). At the early flower bud differentiation stage, the basal region of the bud began to expand and the outer surface grew yellowish brown hairs. Inside the growing flower bud, the spathe-like bracts began to be stratify (Figure 1B). The floral primordium became larger and uplift deformation was observed, although the cells were still closely arranged (Figure 1H). At the petal primordium differentiation stage, the bud grew longer and wider and became distinct from the leaf primordia. Inside the bud were a few layers of spathe-like bracts with scale hairs (Figure 1C). The tip of the developing floral meristem had an undulating surface, indicating the initiation of petal primordium differentiation (Figure 1I). During the stamen primordium differentiation stage, the buds were enlarged and the outer yellowish-brown hairs gradually became denser (Figure 1D). The differentiation region of the inner bud became wider and elongated, forming a hump shade with a smooth tip. The outer cells of the bud meristem were small and compact, while the inner cells were separated from each other. Inside the petal primordia, many rows of small protruding spots were formed around the bottom of the meristem, which were determined to be the differentiating stamen primordia (Figure 1J). At the pistil primordium differentiation stage, the bud volume further increased and the external scale hairs became fluffy (Figure 1E,F). The inner developing bud tip elongated and its base became thick, while its top formed a smooth conical shape. At this stage, the upper region of the meristem formed multiple round bulges, indicating that the pistil primordia had begun to differentiate (Figure 1K). The bulges were retained until the differentiation was complete (Figure 1L).

### 3.2. Differentiating Flower Bud Transcriptome Sequencing, Assembly, and Functional Annotation

Separate transcriptomes were obtained from the five stages of *M. sinostellata* flower bud differentiation, with three biological replicates performed for each stage. A total of 110.56 Gb raw data was obtained during transcriptomic sequencing. The raw data were uploaded to NCBI under the accession numbers SRP129819 and SRR6475480–SRR6475509. After removing the unqualified reads from the raw data, the Q20 was above 97.53% and the GC number was 49.14–51.65% (Table S3). The mapping rate for the assembled transcriptomes was more than 86.08% (Table S4). The N50 length was 1126 bp and the size of the unigenes was generally between 200 bp and 2000 bp, with a mean length of 648 bp (Table S5). After assembly, a total of 52,441 Unigenes were obtained from 15 samples at different developmental stages, which were compared with Swiss-prot [32], Nr [33], KEGG [34], KOG [35], Pfam [36], and GO [37] for annotation and analysis (Table 1, Table S6). In the distribution of species, 59.8% of unigenes could be annotated based on their sequence similarity to unigenes from other species; the species providing the highest number of significant hits were grapevine (*Vitis vinifera*) and date palm (*Phoenix dactylifera)*, which were used to annotate 23.2% and 19.2% of the transcripts, respectively (Figure S2).

### 3.3. Genes Expression in Each Stage of Flower Bud Differentiation

**Figure 2:**
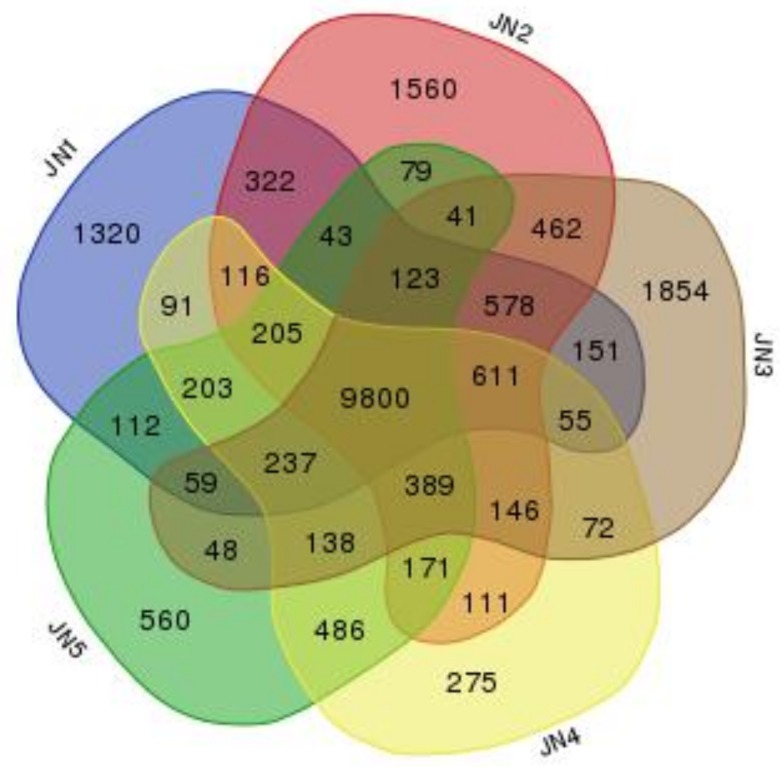
Venn diagram of gene number in the five developmental stages.

The genes number was analyzed between the five stages of flower bud differentiation (Figure 2, Figure S3). The number of genes expressed in the five stages (from undifferentiated to pistil primordium differentiation) was 14,026; 14,757; 14,764; 13,106; and 12,794, respectively. The number of genes specifically expressed in each stage was 1320; 1560; 1854; 275; and 560, respectively (Figure 2). According to the criteria FDR < 0.05, the differentially expressed genes of the three replicates for each floral differentiation stage showed in Table S6, indicating that gene expression changed significantly during *M. sinostellata* flower bud development and that fewer genes were specifically expressed in the late stages of flower bud differentiation.

### 3.4. GO and KEGG Enrichment Analysis of Differentially Expressed Genes

**Figure 3:**
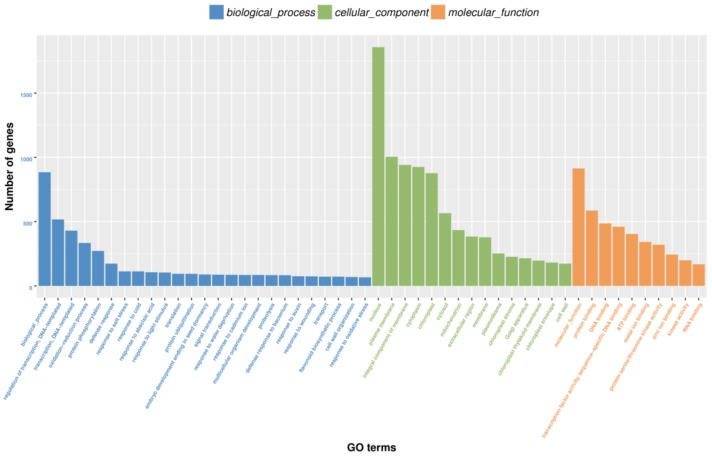
GO function classification in flower bud differentiation differentially expressed genes (DEGs).

**Figure 4:**
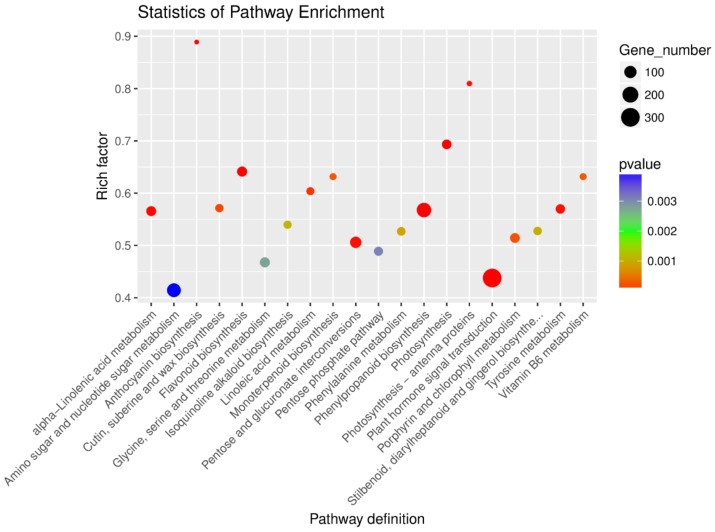
The top 20 of KEGG pathway enrichments in flower bud differentiation DEGs.

The RNA-seq analysis revealed a total of 52,441 genes, of which 11,592 genes were significantly differentially expressed in the five stages (FDR < 0.05). A gene ontology (GO) enrichment analysis of the DEGs revealed a significant enrichment of 371 GO terms throughout the differentiation process (*p* < 0.05) (Table S7). The GO terms were divided into three major categories—i.e., molecular function, cell component, and biological process—as well as 50 minor classes (Figure 3). The DEGs were successfully annotated as members of 135 pathways and the number of significantly enriched KEGG pathways was 36 (*p* < 0.05) (Table S8). Relatively high numbers of genes were annotated as plant hormone signal transduction (map04075; 303 genes), phenylpropanoid biosynthesis (map00940; 155 genes), amino sugar and nucleotide sugar metabolism (map00520; 138 genes), pentose and glucuronate interconversions (map00040; 83 genes), and flavonoid biosynthesis (map00941; 59 genes) pathways, with Rich factors of 0.44, 0.57, 0.41, 0.51, and 0.64, respectively (Figure 4). A Rich factor is the ratio of the number of DEGs annotated with a pathway term relative to the total number of genes annotated with this pathway term. The larger the Rich factor, the greater the enrichment of this KEGG pathway.

### 3.5. DEGs and Transcription Factors Associated with the Five Stages of Flower Bud Differentiation

**Figure 5:**
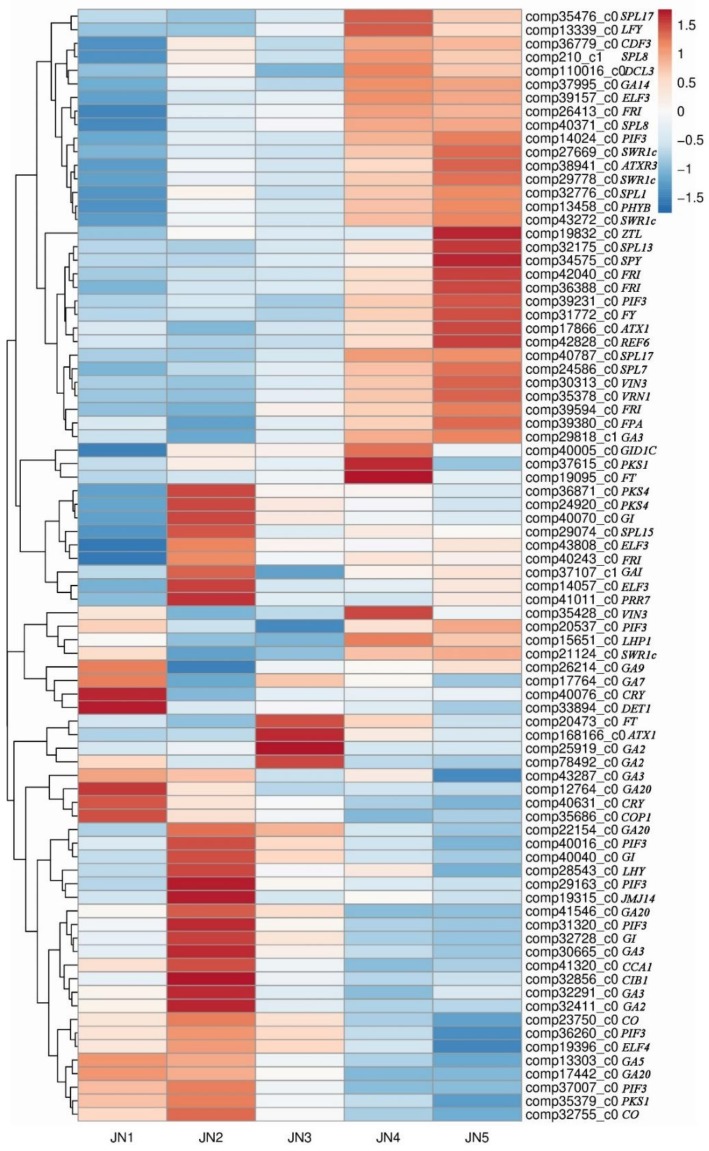
The expression of DEGs putatively related to flowering-pathway genes in *Arabidopsis thaliana.* Comp indicates the prefix of the gene ID. Red and Blue represents up- and down-regulated DEGs, respectively.

We identified 82 significantly differentially expressed *M. sinostellata* genes that were putative homologs of the flower bud differentiation genes in *A. thaliana* (Figure 5)*.* In the circadian rhythm pathway, the expression levels of *CRY* (*CRYPTOCHROME*) decreased and *ELF3* (*EARLY FLOWERING 3*) increased, while the expression of *GI* (*GIGANTEA*) and *CO* (*CONSTANS*) increased during the early stages of *M. sinostellata* floral bud differentiation and decreased in the later stages (Table S9). The expression levels of *FY*, *FPA*, and *SPL* (*SQUAMOSA PROMOTER BINDING PROTEIN-LIKE*) were lower than those of flower bud differentiation. The expression of *FRI* (*FRIGIDA*) increased during flower bud differentiation. A total of 15 DEGs were associated with the gibberellin pathway; however, no consistent changes in the expression of these genes were observed during floral development. The expression of *FT*, an integrator of flowering regulatory signals [3], increased and then decreased as floral differentiation progressed.

**Figure 6:**
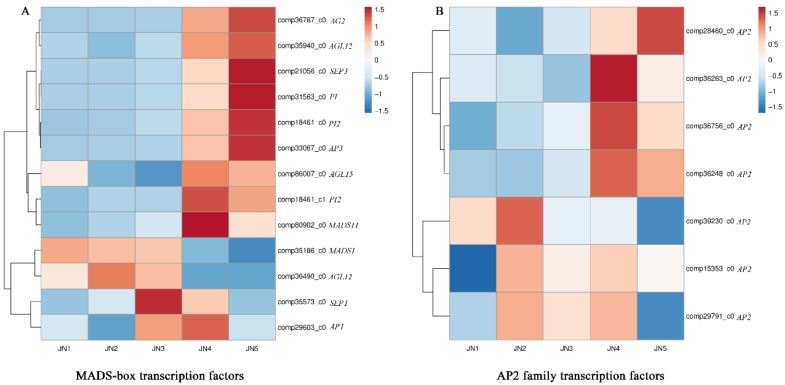
The expression of DEGs putatively encoding MADS-box and *AP2* family transcription factors. (**A**) is the expression of DEGs putatively encoding MADS-box; (**B**) is the expression of DEGs putatively encoding AP2 family transcription factors. Comp indicates the prefix of the gene ID. Red and Blue represents up- and down-regulated DEGs, respectively.

The floral development model is composed of the activities of MADS-box and *AP2* family transcription factors [13,18]. The expression levels of MADS-box and *AP2* family transcription factor-related genes differed between the five stages of floral development (Figure 6). Other genes encoding putative transcription factors were also differentially expressed throughout flower bud differentiation; for example, 36 genes encoding putative MYB transcription factors were differentially expressed during flower bud differentiation (Figure S4), as were 18 *NAC* family genes (Figure S5), 27 *WRKY* genes (Figure S6) and 13 *GATA* genes (Figure S7).

### 3.6. Verification of Relative Gene Expression in Different Stages of Flower Bud Differentiation

**Figure 7:**
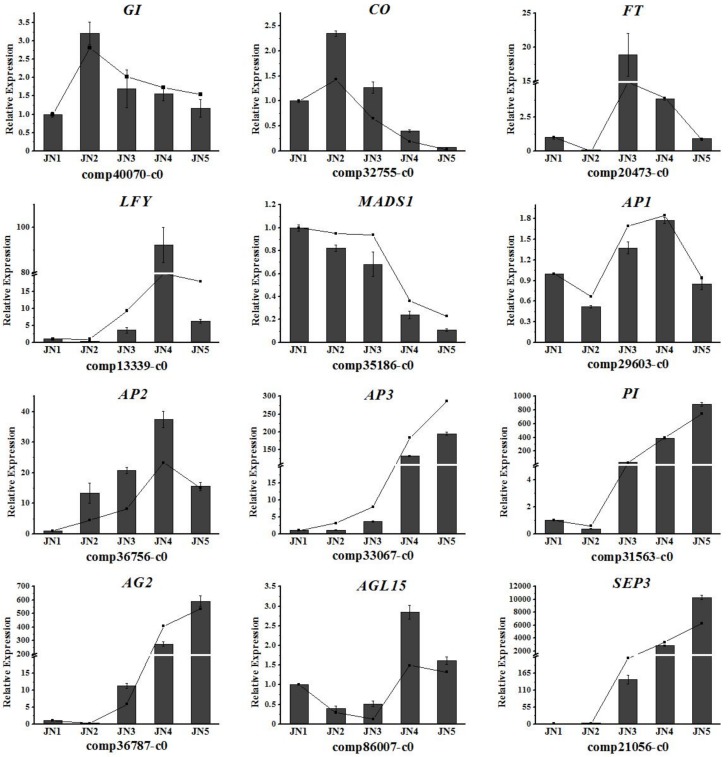
qRT-PCR analysis of the expression 12 DEGs. These 12 putative MADS-box family, *AP2* family and circadian rhythm pathway genes were related to the formation of flower. The undifferentiated stage was used as a control group when calculating the relative expression quantity. Line were from the results of transcriptome data; column charts were from the results of qRT-PCR.

Twelve putative MADS-box family, AP2 family, and circadian rhythm pathway genes were selected from the DEGs: *GI*, *CO*, *FT*, *LFY*, *AP1* (*APETALA1)*, *AP2*, *AP3*, *PI* (*PISTILLATA*), *MADS1*, *AG2* (*AGAMOUS2*), *AGL15* (*AGAMOUS-LIKE-15*), and *SEP3.* The expression patterns of these 12 genes during flower bud differentiation were verified using qRT-PCR (Figure 7) and their expression trends were found to be similar to those obtained by RNA-seq, suggesting that the RNA-seq data reliably reflect the gene expression trends. According to the RNA-seq and qRT-PCR results, *GI*, *CO*, *FT*, and *LFY* expression increased before the onset of flower bud differentiation. *AP1* and *AP2* belong to the A-class genes and their expression levels increased during petal primordium differentiation and decreased gradually at the later stages. The expression levels of *AP3*, *PI*, *AG2*, *AGL15*, and *SEP3* were significantly increased after petal primordium differentiation, which was consistent with the expression levels of B-, C-, and E-class genes. *MADS1* expression decreased during flower bud differentiation, suggesting that low levels of *MADS1* expression induce flower formation from the buds in the early stages of floral development.

## 4. Discussion

Flower bud differentiation is the turning point from vegetative to reproductive growth, which occurs after the plant has had time to accumulate the required nutrients [43,44]. *M. sinostellata* buds take almost a month to develop and the paraffin section staining performed here showed that the flower bud primordia began to elongate and broaden as they differentiated. The floral tissues developed gradually and sequentially, from the outer sepals and petals to the inner stamens and pistils. The observed morphological changes were similar to those of *Liriodendron tulipifera*, *Magnolia championii*, *Magnolia delavayi*, *Magnolia grandiflora*, and *Magnolia paenetalaum* [20]. The flower bud differentiation of *M. sinostellata* could be divided into five stages; the undifferentiated stage, early flower bud differentiation, petal primordium differentiation, stamen primordium differentiation, and pistil primordium differentiation. These five stages are consistent with the flower bud differentiation stages observed in *Ziziphus jujube* [45], *Prunus armeniaca* [46], and *Cerasus tianschanica* [47].

In this study, transcriptome information was obtained by the high-throughput sequencing of flower buds at the five stages of flower bud differentiation. We identified 11,592 genes that were significantly differentially expressed during differentiation (FDR < 0.05). The numbers of genes specifically expressed in the undifferentiated, early flower bud differentiation and petal primordial differentiation stages were 1320, 1560, and 1854, respectively, while the numbers of genes specifically expressed in the stamen primordium differentiation and pistil primordium differentiation stages were only 275 and 560, respectively. This indicated many genes regulate flower bud differentiation and that the majority were involved in the early stages of floral development [48]. The GO annotations in the DEGs focused mainly on the regulation of biological processes, nucleus, and molecular function. The KEGG annotations showed that the DEGs were highly enriched in plant hormone signal transduction, indicated that many endogenous hormones were involved in the flowering of *M. sinostellata*, not just gibberellins at this work [49]. Many DEGs were related to pentose, glucuronate interconversions, amino sugar, and nucleotide sugar metabolism suggesting that sugar metabolism may be another important pathway to regulate the flowering of *M. sinostellata* [50]. In addition, DEGs were also enriched in circadian rhythm and flavonoid biosynthesis. These indicated that the plant received light signals which induced the transcriptional regulation of a variety of pathways and led to the accumulation of flavonoids in floral tissues [51].

The putative MYB, NAC, WRKY, and GATA transcription factors were differentially regulated during floral differentiation in *M. sinostellata*. These transcription factors are known to be involved in plant growth and development. The MYBs participate in plant secondary metabolism, responding to phenylalanine metabolism, signals from the external environment and phytohormones and play major roles in the flavonoid and pigment biosynthesis metabolic pathways [52,53]. The MYB transcription factors are also involved in the signal transduction of gibberellin, which regulates *LFY* expression to control flowering [54,55]. The NAC transcription factors are also involved in a variety of developmental and physiological processes and play important roles in hormone signal transduction, bud apical meristem formation, and the maintenance of flower development and morphology [56]. The WRKYs also play a role in the transduction of hormone signals during the growth and development of plants [57]. The GATA transcription factors have been shown to affect the regulation of floral development in response to the transmission of light signals [58,59]. The effects of these transcription factors are consistent with the changes in their expression observed during flower bud differentiation.

Some of the genes involved in the six major pathways regulating *Arabidopsis* flower formation were also found to be differentially expressed in flower bud differentiation in *M. sinostellata*. The red-light and blue-light receptor genes, *PHY* and *CRY*, were differentially expressed; *CRY* expression was decreased, while the expression of *GI* was increased, with later increases observed in the expression of *CO*, which promotes *M. sinostellata* flowering. *LFY*, which is expressed in the meristem, is considered to be the switch that determines flower development [60,61]. The expression of *LFY* was significantly altered during flower bud differentiation in *M. sinostellata*, indicating that *LFY* also plays a key role in the development of magnolia flowers. Our transcriptome sequencing results indicated that many different genes and pathways are involved in flower bud differentiation. FLC is a negative regulator of flowering involved in the vernalization and autonomous pathways [62]. *FY* and *FPA* act in the autonomous pathway to promote the expression of *FT* by inhibiting *FLC*, while in the vernalization pathway, *FRI* promotes *FLC* expression to inhibit flowering [41,63]. *FLC* was not found amongst the expressed genes of flower bud differentiation in *M. sinostellata*, but the expression levels of *FY*, *FPA*, *FRI*, and *FT* were all elevated, indicating that *FY*, *FPA*, and *FRI* may have other ways of promoting flowering. The expression of *SPL,* associated with the senescence pathway, increased during flower development, suggesting that this gene might also promote *AP1* and *LFY* expression to induce flowering in *M. sinostellat*a [64,65]. Studies have shown that gibberellin plays an important role in flower bud differentiation [65,66]. A total of 15 DEGs were found to be related to gibberellin-pathway genes; however, their expression did not follow any obvious patterns and their mechanism of action requires further study. These results suggest that flower bud differentiation in *M. sinostellata* is not the result of a single category of gene function, but rather the result of many genetic interactions.

The various pathways involved in flower induction eventually converge on the floral meristem, where the MADS-box and *AP2* family transcription factors play a role in flower development [67] (Figure S8). According to our RNA-seq and qRT-PCR results, the expression levels of *AP1*, a flowering marker gene [68], began to increase when the petal primordium differentiated. The related gene *AP2* is also an A-class gene and has a more pronounced flowering-development expression pattern. *AP3* and *PI* are B-class genes and were found to have lower levels of expression prior to stamen differentiation, after which their expression increased dramatically [69]. The expression patterns of the C-class genes were found to contrast with those of the A-class genes, indicating the possible existence of negative feedback regulation between the two groups, which was previously reported in the classic ABC model [70].

## 5. Conclusions

In this study, we characterized the morphological changes of flower bud differentiation in *M. sinostellata* and divided the differentiation period into five distinct stages. The expression patterns of genes during these different stages were determined using the transcriptome sequencing of flower buds during their differentiation and those specifically involved in flower bud development were identified. The results of a qRT-PCR analysis enabled us to identify the transcriptional changes of genes involved in the differentiation of the different floral organs throughout flower development. These results represent a first step towards illuminating the molecular mechanisms of flower development in *Magnolia* sp. and provide abundant genomic resources and new candidate genes for the study of flowering regulation, as well as highlighting possible molecular breeding targets in this plant species.

## Figures and Tables

**Table 1:** Functional unigene annotations

	Gene Number	Unigenes Annotated Using These Databases					
		Nr	Pfam	KEGG	KOG	Swiss-Prot	GO
Unigenes	52,441	25,531	18,736	8612	20,977	15,850	14,055
Percentage	100%	48.69%	35.73%	16.42%	40.00%	30.22%	26.80%

## Supplementary Materials

The following are available online at http://www.mdpi.com/2073-4425/9/4/212/s1, Table S1: Primers used to analyze the expression of the flower bud differentiation-related genes and the reference gene; Table S2: Analysis of internal reference genes expression stability and ranking by BestKeeper software. Table S3: Sequencing data quality preprocessing results; Table S4: Mapping rates of sequencing data assembly; Table S5: Sequencing data assembly results; Table S6: All genes in five stages; Table S7: GO enrichment of the DEGs; Table S8: KEGG pathway enrichment of the DEGs; Table S9: The 82 DEGs related to flowering-pathway genes in *Arabidopsis* sp.; Figure S1:Analysis of internal reference genes expression stability by geNorm software. The smaller the M value, the better the stability. Figure S2: The distribution of species used to annotate the transcripts; Figure S3: Pearson correlation between samples; Figure S4: MYB transcription factor expression levels during flower bud differentiation.; Figure S5: NAC transcription factor expression levels during flower bud differentiation.; Figure S6: WRKY transcription factor expression levels during flower bud differentiation.; Figure S7: GATA transcription factor expression levels during flower bud differentiation. Figure S8: The pathway of flowering in *M*. *sinostellata*. Se: Scales; Pe: Petal primordium; St: Stamen primordium; Pi: Pistil base; Yellow is B-class genes, Purple is A-class gene, and Red is C- and E-class genes.
