# Supplementary figures and images for: Transcriptomic Analysis of Flower Bud Differentiation in Magnolia sinostellata
Source: Genes (Basel). 2018 Apr 16;9(4):212. doi: 10.3390/genes9040212 (PMC5924554; doi:10.3390/genes9040212)

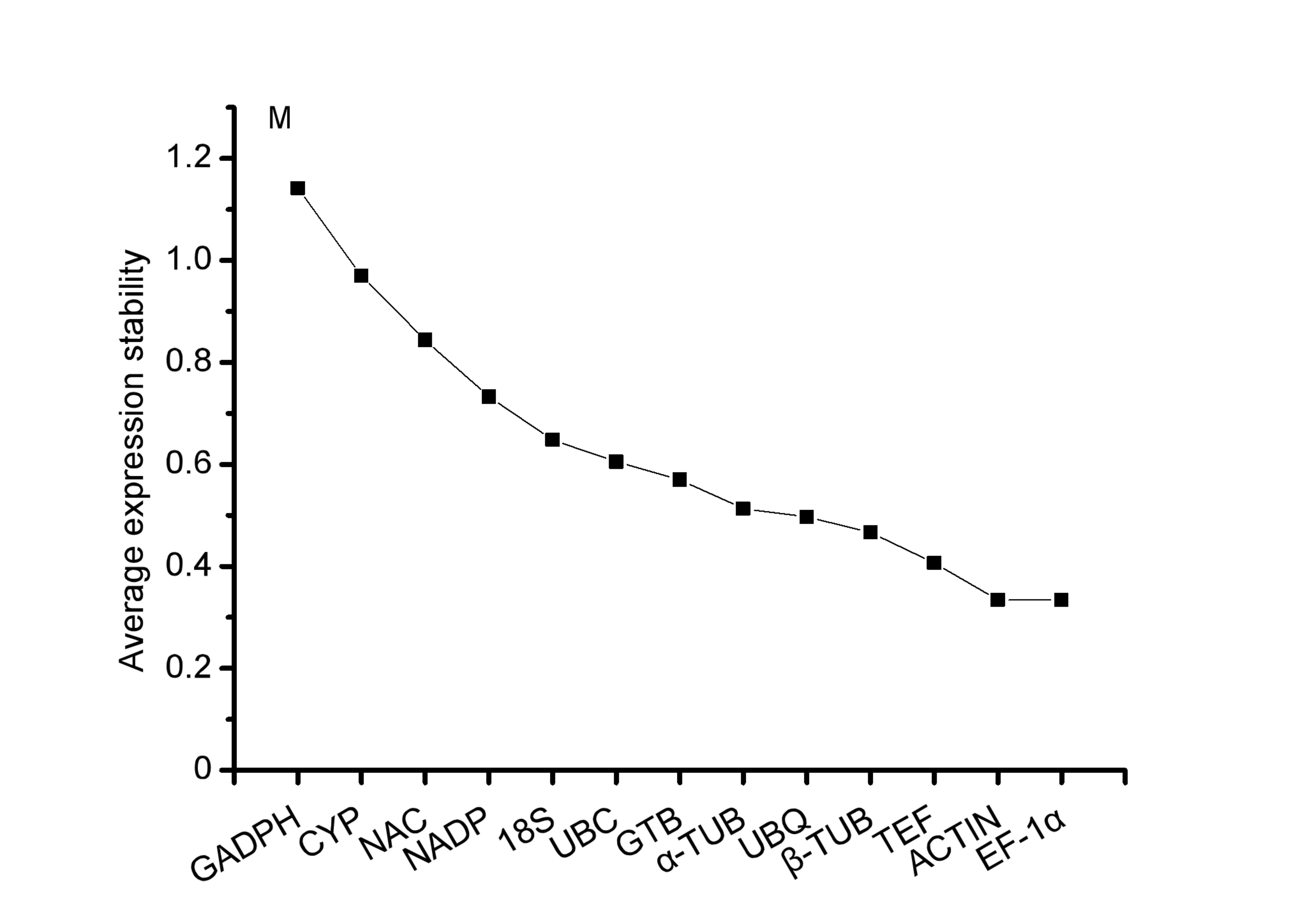

Supplement: Supplementary file 1 [file genes-09-00212-s001.zip › Figure S1.png]
